# *Ixodes scapularis* salivary gland microRNAs are differentially expressed during Powassan virus transmission

**DOI:** 10.1038/s41598-019-49572-5

**Published:** 2019-09-11

**Authors:** Meghan E. Hermance, Steven G. Widen, Thomas G. Wood, Saravanan Thangamani

**Affiliations:** 1 SUNY Center for Environmental Health and Medicine, SUNY Upstate Medical University, Syracuse, NY United States; 2 Institute for Global Health and Translational Science, SUNY Upstate Medical University, Syracuse, NY United States; 3 Department of Microbiology and Immunology, SUNY Upstate Medical University, Syracuse, NY United States; 4 Department of Biochemistry and Molecular Biology, University of Texas Medical Branch, Galveston, TX United States

**Keywords:** Microbiology, Cellular microbiology

## Abstract

Successful tick feeding is facilitated by an assortment of pharmacologically-active factors in tick saliva that create an immunologically privileged micro-environment in the host’s skin. Through a process known as saliva-assisted transmission, bioactive tick salivary factors modulate the host environment, promoting transmission and establishment of a tick-borne pathogen. This phenomenon was previously demonstrated for Powassan virus (POWV), a North American tick-borne flavivirus that is the causative agent of a severe neuroinvasive disease in humans. Here, we sought to characterize the *Ixodes scapularis* salivary gland microRNAs (miRNAs) expressed during the earliest period of POWV transmission to a mammalian host. POWV-infected and uninfected *I*. *scapularis* females were fed on naïve mice for 1, 3, and 6 hours, and Illumina next generation sequencing was used to characterize the salivary gland miRNA expression profiles of POWV-infected versus uninfected ticks. 379 salivary miRNAs were detected, of which 338 are reported here as putative novel *I*. *scapularis* miRNAs. 35 salivary gland miRNAs were significantly up-regulated and 17 miRNAs were significantly down-regulated in response to POWV infection. To investigate the potential role of salivary gland miRNAs in POWV replication *in-vitro*, we transfected miRNA inhibitors into VeroE6 cells to profile temporal POWV replication in mammalian cells. Together, the small RNA sequencing data and the *in vitro* miRNA inhibition assay suggest that the differentially expressed tick salivary miRNAs could act in regulating POWV replication in host tissues.

## Introduction

*Ixodes scapularis* ticks, otherwise known as the Deer tick, are vectors for several bacterial, protozoal, and viral pathogens in North America. One such pathogen is Powassan virus (POWV), an emerging tick-borne flavivirus (TBFV) that can result in severe neurological symptoms in humans with case fatality rates ranging from 10–15% in most reports^1–5^. Long-lasting neurological sequelae, including hemiplegia, recurrent acute headaches, muscular atrophy, and memory loss, have been documented in 50% of survivors^1,2,4,5^. In recent years, an expanded geographic range of *Ixodes* species ticks has been documented in the Continental United States coupled with an increase in reported POWV human disease cases^6–9^.

*I*. *scapularis* ticks transmit POWV via saliva to the skin of a vertebrate host during blood meal acquisition. In as little as minutes to a few hours of tick feeding, a TBFV can be transmitted to the host by a single *Ixodes* species tick^10–12^. Ticks are distinct from other blood-feeding arthropods in that they attach to a vertebrate host for extended periods of time as they ingest large amounts of blood. In order to acquire a blood meal, ticks must overcome the host’s immune and hemostatic defenses; consequently, successful tick feeding is facilitated by a complex collection of bioactive salivary factors secreted into the feeding pool on the vertebrate host’s skin, ultimately enabling the tick to remain attached and continue feeding. In a process known as saliva-assisted transmission (SAT), pharmacologically active molecules from tick saliva modulate various host defense mechanisms, creating an environment that is more favorable for pathogen transmission and establishment^13–16^. SAT has been demonstrated for several TBFVs, including POWV and the closely related tick-borne encephalitis virus (TBEV)^17,18^. We previously showed that the presence of unfed *I*. *scapularis* salivary gland extract (SGE) enhances the transmission of POWV and accelerates the disease progression for naive BALB/c mice infected with a low dose of POWV^17^. Currently, no *in vivo* data has implicated a specific tick salivary protein or nucleic acid for SAT of any TBFV. We believe that a combination of salivary factors acting synergistically are responsible for enhancement of TBFV transmission^19^.

MicroRNAs (miRNAs) are short non-coding RNAs, approximately 22 nucleotides in length, that regulate gene expression at the post-transcriptional level. miRNAs can induce translational inhibition or stimulate targeted degradation of messenger RNAs (mRNAs) via binding to the 3’ UTR region of target mRNAs^20^. A single miRNA may bind to many different mRNA targets, while a given mRNA target may be regulated by multiple miRNAs^21^. This phenomenon of combinatorial regulation highlights the importance of miRNAs in gene regulatory networks^21^. miRNAs are involved in regulating a variety of arthropod physiological processes, including development and blood feeding^22^. A limited number of studies have examined tick miRNA expression patterns with reports of evolutionary-conserved miRNAs expressed in all life stages of *Rhipicephalus microplus*^23^, differentially expressed miRNAs in fed versus unfed *Haemaphysalis longicornis* salivary glands^24^, gender-specific *Rhipicephalus sanguineus* miRNAs^25^, lipopolysaccharide-induced differential expression of *Rhipicephalus haemaphysaloides* miRNAs^26^, and saliva-specific miRNAs expressed in *Ixodes ricinus*^27^. Recently, in what is currently the only *in vivo* functional study conducted for a tick-specific miRNA, miR-275 was shown to target Vitellogenin-2 in *H*. *longicornis*, regulating blood digestion, ovary development, and egg mass^28^.

Despite miRNA publications for five tick species, the miRBase database currently contains cataloged miRNAs for only two tick species, with 49 annotated *I*. *scapularis* miRNAs and 24 annotated *R*. *microplus* miRNAs (miRBase 22, http://www.mirbase.org/). There are slightly more mosquito miRNAs annotated in miRBase, with 156 *Aedes aegypti* miRNAs, 76 *Culex quinquefasciatus* miRNAs, and 131 *Anopheles gambiae* miRNAs. While there are currently more mosquito miRNA publications than tick miRNA publications^20^, little miRNA profiling has been conducted in the salivary glands/saliva of either mosquitoes or ticks. Two studies have examined the miRNA expression profiles in uninfected tick salivary glands of *R*. *(Boophilus) microplus* and *H*. *longicornis*^23,24^, and one study has profiled the miRNAs expressed in uninfected *I*. *ricinus* saliva and salivary glands^27^. Only a single publication characterizes arthropod saliva miRNA profiles in response to virus infection. Exogenous miRNAs were identified in *A*. *aegypti* and *Aedes albopictus* saliva, and *in vitro* data suggests that several of these miRNAs play important roles in regulating Chikungunya virus (CHIKV) infection^29^. At present, no study has examined tick saliva/salivary gland miRNAs in relation to virus infection.

In the current study, we sought to examine the effects of POWV infection on salivary gland miRNA expression profiles and their regulation during POWV transmission to a mammalian host. Next generation sequencing was performed on salivary glands of POWV-infected ticks during the earliest period of blood feeding and POWV transmission. To our knowledge, this is the first study to examine tick salivary gland miRNA expression profiles in relation to virus infection.

## Results

### Profile characteristics of small RNA libraries

POWV-infected and uninfected *I*. *scapularis* adult females were fed on naïve mice for 1, 3, and 6 hours (1 tick per mouse), and tick salivary glands were dissected at each time point. RNA (including small RNA molecules < ~200 nucleotides) was extracted from the salivary glands of every fed tick, and a separate small RNA library was prepared for each pair of tick salivary glands (21 small RNA libraries total). POWV infection was confirmed in individual tick salivary glands by qPCR. The average POWV titers in infected tick salivary glands after 1 hour, 3 hours, and 6 hours of tick feeding was 4.15 × 10^6^ focus forming units (FFU)/µg RNA, 1.20 × 10^6^ FFU/µg RNA, and 2.46 × 10^6^ FFU/µg RNA, respectively (Table 1).

**Table 1:** Viral loads in salivary glands of ticks confirmed by qPCR.

	POWV-infected tick salivary glands (SGs)	Uninfected tick salivary glands (SGs)
1 hour of tick feeding	n = 4; Av. titer = 4.15 × 10^6^ FFU/µg RNA	n = 3; Av. titer = 0 FFU/µg RNA
3 hours of tick feeding	n = 4; Av. titer = 1.20 × 10^6^ FFU/µg RNA	n = 3; Av. titer = 0 FFU/µg RNA
6 hours of tick feeding	n = 4; Av. titer = 2.46 × 10^6^ FFU/µg RNA	n = 3; Av. titer = 0 FFU/µg RNA

Four biological replicates were included for each of the 3 POWV-infected tick feeding time points (i.e. 4 pairs of POWV-infected tick salivary glands were harvested at 1 hour resulting in 4 individual small RNA libraries, 4 pairs of infected salivary glands were harvested at 3 hours resulting in 4 small RNA libraries, and 4 pairs of infected salivary glands were harvested at 6 hours resulting in 4 small RNA libraries). Three biological replicates were included for each of the 3 uninfected tick feeding time points (i.e. 3 pairs of uninfected tick salivary glands were harvested at 1 hour resulting in 3 individual small RNA libraries, 3 pairs of uninfected salivary glands were harvested at 3 hours resulting in 3 small RNA libraries, and 3 pairs of uninfected salivary glands were harvested at 6 hours resulting in 3 small RNA libraries). Four biological replicates (instead of 3) were included for each of the POWV-infected tick feeding time points to better account for any potential variation of POWV titers between salivary gland samples. In total, 21 small RNA libraries were included in this study.

The 21 small RNA libraries were sequenced via Illumina small RNA high-throughput sequencing to identify short non-coding RNAs in the tick salivary gland samples. A combined total of 526.6 million small RNA raw reads were detected in all *I*. *scapularis* salivary gland libraries, including both POWV-infected and uninfected salivary glands. From the POWV-infected 1 hour, 3 hour, and 6 hour fed salivary gland libraries, 104.8 million, 146.0 million, and 97.9 million reads with adapter sequences were acquired, respectively. Of the total reads processed in different POWV-infected salivary gland libraries, 0.22–6.95% were discarded. This resulted in 103.9 million, 142.8 million, and 97.9 million clean reads with passing filters for the POWV-infected 1 hour, 3 hour, and 6 hour fed salivary gland libraries, respectively. Likewise, from the uninfected 1 hour, 3 hour, and 6 hour fed salivary gland libraries, 62.8 million, 49.9 million, and 61.7 million reads with adapter sequences were acquired, respectively. Of the total reads processed in different uninfected salivary gland libraries, 0.23% - 2.96% were discarded. This resulted in 62.8 million, 49.7 million, and 61.0 million clean reads with passing filters for the uninfected 1 hour, 3 hour, and 6 hour fed salivary gland libraries, respectively.

**Figure 1:**
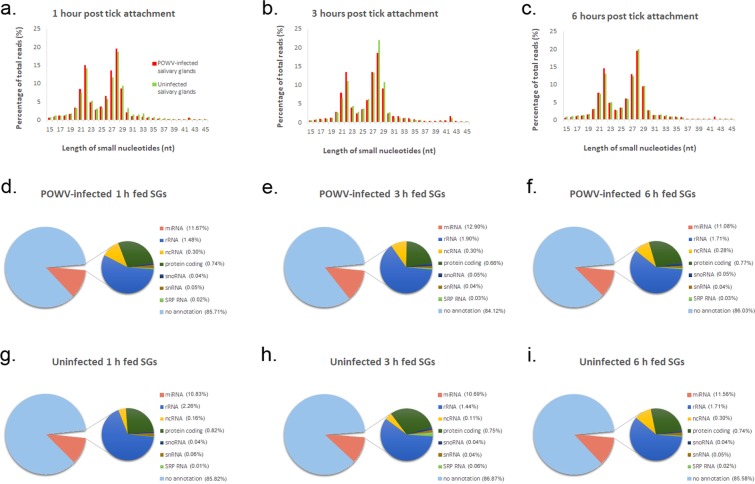
Summary of next generation sequencing of tick-derived small RNAs obtained from POWV-infected and uninfected *Ixodes scapularis* salivary glands at 1 hour, 3 hours, and 6 hours post tick attachment. Salivary glands were dissected from POWV-infected female *I*. *scapularis* ticks and uninfected female ticks that were fed on mice for 1, 3, or 6 hours. RNA was extracted from the salivary glands, and 21 small RNA libraries were generated then subjected to Illumina small RNA sequencing. (**a**–**c**) Size distribution of tick-derived small RNA reads with passing filters mapped to the *I*. *scapularis* genome. (**d**–**f**) Summary of tick-derived reads that match various small RNA categories from POWV-infected tick salivary glands at 1 hour, 3 hours, and 6 hours post tick attachment. (**g**–**i**) Summary of tick-derived reads that match various small RNA categories from uninfected tick salivary glands at 1 hour, 3 hours, and 6 hours post tick attachment.

The predominant size distribution of these small RNA reads was 20–30 nucleotides (nt). Based on the size distribution of the clean reads with passing filters at each experimental time point, a bimodal size distribution was detected, with one peak around 21–23 nt representing miRNAs and another peak around 26–29 nt likely representing piwi-interacting RNAs (piRNA) (Fig. 1). For the 1 hour POWV-infected salivary gland libraries, the highest percentage of clean reads (19.43% of library) were 28 nt long, followed by 14.90% that were 22 nt (Fig. 1a). Similarly, for the 1 hour uninfected salivary gland libraries, 28 nt reads were most abundant (18.51%), followed by 22 nt reads (14.05%). For all infected and uninfected 3 hour salivary gland libraries, the most abundant reads were 28 nt, followed by 27 nt, then 22 nt. Specifically, 28 nt reads (18.42%), 27 nt reads (13.30%), and 22 nt reads (13.28%) were most abundant for the 3 hour POWV-infected libraries, while 28 nt reads (21.80%), 27 nt reads (13.21%), and 22 nt reads (10.95%) were most abundant for the 3 hour uninfected libraries (Fig. 1b). Similar to the read abundance pattern displayed for the 1 hour libraries, 28 nt reads (19.34%) followed by 22 nt reads (14.48%) were most abundant for the 6 hour POWV-infected libraries, and 28 nt reads (19.90%) followed by 22 nt reads (13.01%) were most abundant for the 6 hour uninfected libraries (Fig. 1c). For all three experimental time points, the peaks at 21 and 22 nt were greater for the POWV-infected salivary glands than the uninfected salivary glands, suggesting that miRNAs are more abundant and potentially more diverse in the POWV-infected salivary glands versus the uninfected salivary glands (Fig. 1a–c).

**Figure 2:**
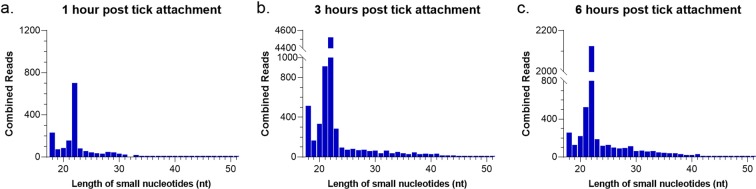
Size distributions of virus-derived small RNAs. Size distribution of mappable reads to the POWV genome (POWV LB Strain, NCBI Reference Sequence: NC_003687.1) in small RNA libraries of POWV-infected female *Ixodes scapularis* tick salivary glands after (**a)** 1 hour, (**b)** 3 hours, and (**c)** 6 hours of tick attachment.

For each experimental time point and infection condition, clean reads were matched to various categories of small noncoding RNAs, including rRNA, snoRNA, and snRNA (Table 2 and Fig. 1d–i). Additionally, small RNA reads were mapped to the POWV genome (POWV LB Strain, NCBI Reference Sequence: NC_003687.1). In total, 1746, 7759, and 4655 reads obtained from the POWV-infected 1 hour, 3 hour, and 6 hour salivary gland libraries, respectively, mapped to the POWV genome. 22 nt reads were most abundant for the virus-derived small RNAs across all three time points (Fig. 2). 40.1%, 58.3%, and 45.6% of virus-derived small RNAs were 22 nt in length for the 1, 3, and 6 hour time points, respectively. Here, the predominance of 22 nt virus-derived small RNAs from the small RNA libraries of POWV-infected, fed *I*. *scapularis* tick salivary glands supports previous findings where POWV lineage II-derived small RNAs were predominantly 22 nt in length in the whole bodies of *I*. *scapularis* larvae, nymphs, and adults^30^.

**Table 2:** Summary of reads that match various small RNA categories from POWV-infected and uninfected *Ixodes scapularis* salivary glands at 1 hour, 3 hours, and 6 hours post tick attachment.

	POWV-infected 1 h fed SGs (%)	POWV-infected 3 h fed SGs (%)	POWV-infected 6 h fed SGs (%)	Uninfected 1 h fed SGs (%)	Uninfected 3 h fed SGs (%)	Uninfected 6 h fed SGs (%)
Total	99207888.3	136723740.2	93519700.7	58979067.2	47335401.3	56582354.4
	(100)	(100)	(100)	(100)	(100)	(100)
miRNA	11574275.0	17637661.0	10366406.0	6387822.0	5059406.0	6541786.0
	(11.67)	(12.90)	(11.08)	(10.83)	(10.69)	(11.56)
rRNA	1464337.2	2595423.1	1599344.2	1330879.4	682997.0	965791.2
	(1.48)	(1.90)	(1.71)	(2.26)	(1.44)	(1.71)
ncRNA	298719.4	408962.5	259681.0	94839.5	50683.5	172379.5
	(0.30)	(0.30)	(0.28)	(0.16)	(0.11)	(0.30)
protein coding	732385.7	902846.9	724128.8	482133.9	355257.3	417336.5
	(0.74)	(0.66)	(0.77)	(0.82)	(0.75)	(0.74)
snoRNA	36336.3	73692.9	48337.3	21871.8	19524.0	20764.3
	(0.04)	(0.05)	(0.05)	(0.04)	(0.04)	(0.04)
snRNA	46774.2	48746.6	36351.7	37950.3	18053.7	30411.5
	(0.05)	(0.04)	(0.04)	(0.06)	(0.04)	(0.05)
SRP RNA	21486.5	37639.2	26240.7	7447.3	28996.8	11974.4
	(0.02)	(0.03)	(0.03)	(0.01)	(0.06)	(0.02)
no annotation	85033574.0	115018768	80459211.0	50616123.0	41120483.0	48421911.0
	(85.71)	(84.12)	(86.03)	(85.82)	(86.87)	(85.58)

### Identification of novel tick miRNAs

**Figure 3:**
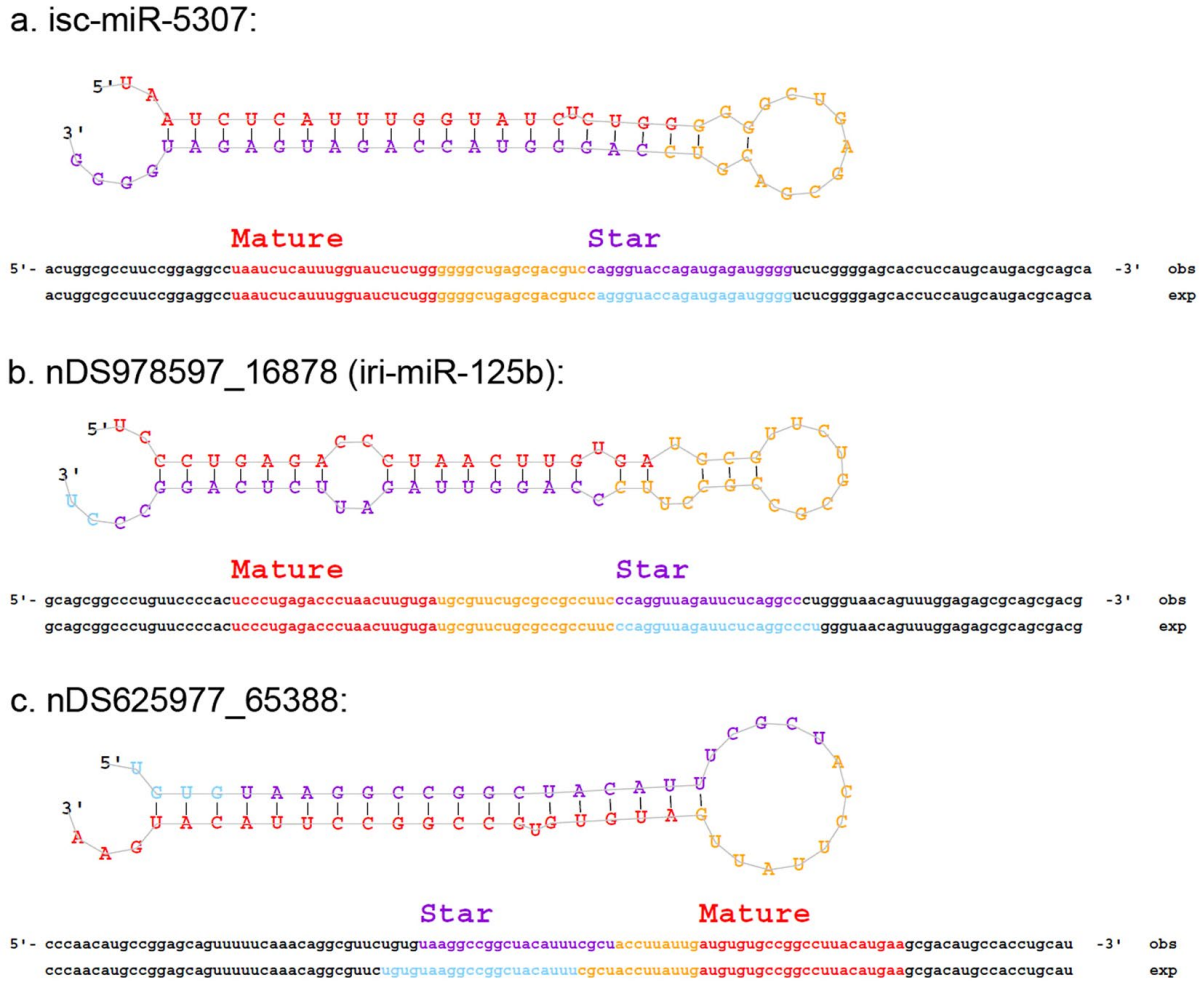
Predicted stem-loop structures of highly expressed microRNAs. The miRDeep2 software was used to identify potential miRNA precursors based on nucleotide length, star sequence, stem-loop folding, and homology to the *I*. *scapularis* reference genome. Figures (**a)** isc-miR-5307 (**b)** nDS978597_16878 and (**c)** nDS625977_65388 show the predicted stem-loop structures, star, and mature sequences of highly expressed miRNAs in *I*. *scapularis* salivary glands.

The miRDeep2 prediction software package was used to identify potential miRNA precursors from the NGS data. The software aligns the reads to the *I*. *scapularis* reference genome and looks for locations where potential miRNA reads stack up. The regions immediately surrounding the mapped reads are examined for miRNA biogenesis features, including mature miRNA, Star and precursor reads, and stem-loop folding properties. Essentially, the miRDeep2 program models the miRNA biogenesis pathway, using a probabilistic algorithm to score compatibility of the position and frequency of NGS reads with the secondary structure of the miRNA precursor^31^. A total of 379 miRNAs were detected in the salivary gland libraries, of which 338 are reported here as potential novel *I*. *scapularis* miRNAs, and 41 miRNAs matched a known, previously annotated miRBase *I*. *scapularis* mature miRNA (Table 3). Supplemental Table 1 includes the consensus mature, star, and precursor sequence, the total read counts, and the miRDeep2 score and probability for every miRDeep2-predicted miRNA with a miRDeep2 score of 4 or greater. The stem-loop structure, the star sequence and premature miRNA sequence are shown for a select few miRNAs that were highly expressed in *I*. *scapularis* salivary glands, including isc-miR-5307, nDS978597_16878, and nDS625977_65388 (Fig. 3).

**Table 3:** miRDeep2 predicted miRNAs that match a known miRBase *I*. *scapularis* miRNA.

miRDeep2 predicted miRNAs that match a known miRBase I. scapularis miRNA					
isc-bantam	isc-miR-153	isc-miR-275	isc-miR-307	isc-miR-5307	isc-miR-7
isc-miR-1	isc-miR-184	isc-miR-276	isc-miR-315	isc-miR-5308	isc-miR-71
isc-miR-10	isc-miR-1993	isc-miR-278	isc-miR-317	isc-miR-5309	isc-miR-79
isc-miR-100	isc-miR-2001	isc-miR-279	isc-miR-375	isc-miR-5310	isc-miR-8
isc-miR-12	isc-miR-219	isc-miR-2a	isc-miR-3931	isc-miR-5312	isc-miR-87
isc-miR-133	isc-miR-252b	isc-miR-2b	isc-miR-5305	isc-miR-5314	isc-miR-96
isc-miR-137	isc-miR-263a	isc-miR-305	isc-miR-5306	isc-miR-5315

### Expression profiles of miRNAs in POWV-infected and uninfected tick salivary glands

**Figure 4:**
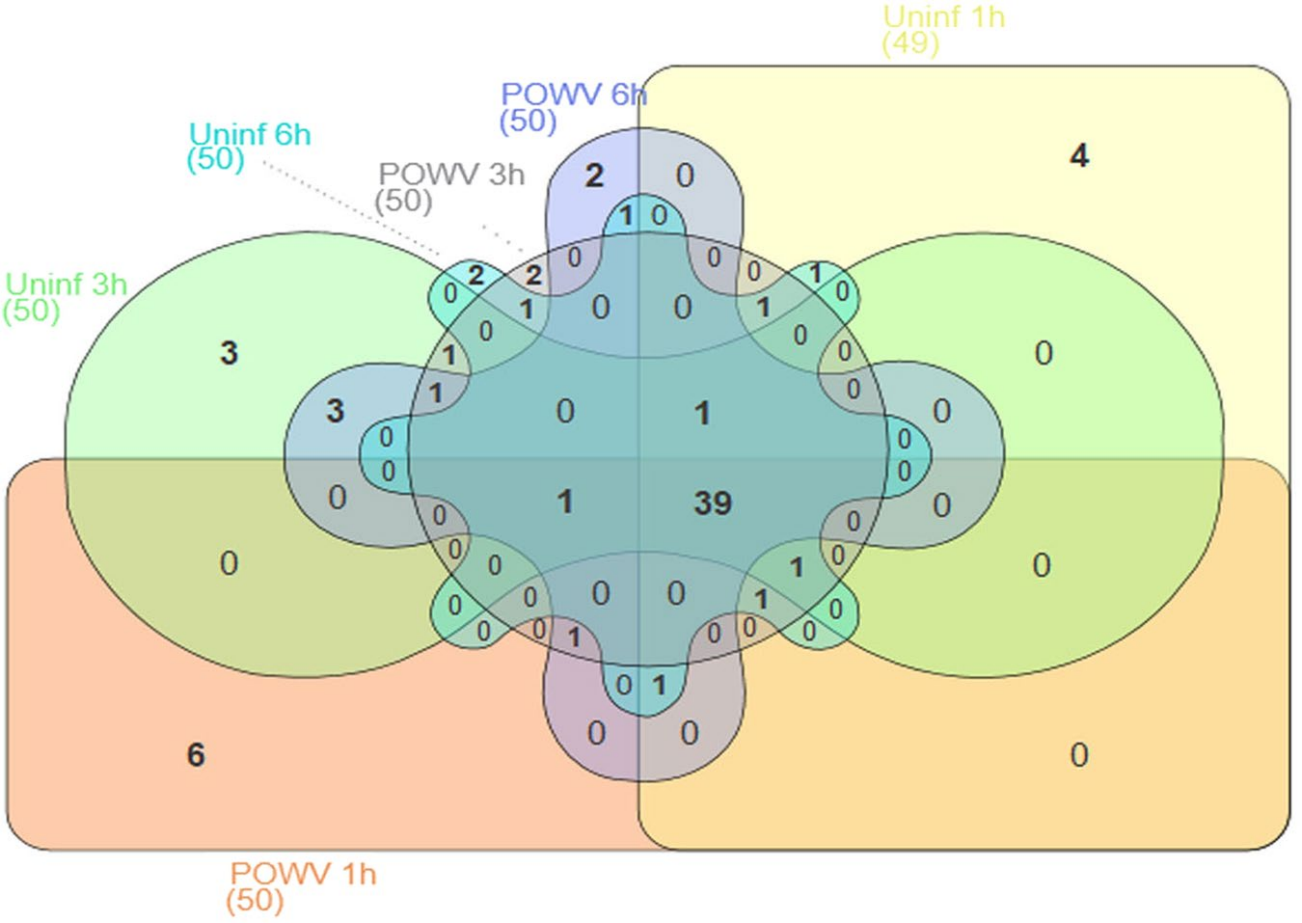
Overlap between the top 50 most abundant miRNAs expressed in POWV-infected 1 hour, 3 hour, and 6 hour fed *Ixodes scapularis* salivary glands, and uninfected 1 hour, 3 hour, and 6 hour fed *I*. *scapularis* salivary glands. In each experimental group, normalized read counts were totaled for every miRNA to generate a list of the top 50 most abundant miRNAs. The online tool, InteractiVenn, was used to generate an Edwards Venn diagram^32^.

For each experimental time point (1 hour, 3 hours, or 6 hours of tick feeding) and infection condition (POWV-infected or uninfected tick salivary glands), the normalized read counts were totaled for every miRNA to generate a list of the top 50 most abundant miRNAs (Table 4). In this text and in the corresponding figures, any miRDeep2-predicted novel miRNA that does not match an already annotated *I*. *scapularis* miRNA is denoted with “nDS” at the beginning of the identifier. The online tool, InteractiVenn, was used to generate an Edwards Venn diagram representing the 50 most abundant miRNAs expressed for each time point and infection condition^32^. Among the lists of top 50 expressed miRNAs, 39 were shared between all 6 experimental groups (Fig. 4). 28 of the 39 shared miRNAs matched a known, previously annotated miRBase *I*. *scapularis* mature miRNA and 5 matched a previously annotated *I*. *ricinus* miRNA, while the remaining 6 miRNAs were predicted to be novel by miRDeep2. Since these 39 shared miRNAs were highly expressed in the uninfected as well as in the POWV-infected tick salivary glands after 1, 3, and 6 hours of tick feeding, the expression of these miRNAs is likely to be independent of POWV infection of the salivary glands.

**Table 4:** The top 50 most abundant miRNAs in each experimental group.

POWV-infected, 1 h fed SGs	POWV-infected, 3 h fed SGs	POWV-infected, 6 h fed SGs	Uninfected, 1 h fed SGs	Uninfected, 3 h fed SGs	Uninfected, 6 h fed SGs
isc-miR-375	isc-miR-5307	isc-miR-375	isc-miR-375	isc-miR-375	isc-miR-375
isc-miR-5307	isc-miR-375	isc-miR-5307	isc-miR-5307	isc-miR-5307	isc-miR-100
isc-miR-100	isc-miR-100	isc-miR-100	isc-miR-100	isc-miR-100	isc-miR-5307
isc-bantam	isc-miR-279	isc-miR-279	isc-bantam	isc-bantam	isc-bantam
isc-miR-279	isc-bantam	isc-bantam	isc-miR-279	isc-miR-279	isc-miR-279
isc-miR-71	isc-miR-71	isc-miR-71	isc-miR-71	isc-miR-71	isc-miR-71
isc-miR-252b	isc-miR-252b	isc-miR-252b	isc-miR-252b	isc-miR-252b	isc-miR-252b
isc-miR-10	isc-miR-1	isc-miR-275	isc-miR-275	isc-miR-10	isc-miR-1
isc-miR-1	isc-miR-10	isc-miR-10	isc-miR-10	isc-miR-275	isc-miR-275
isc-miR-275	isc-miR-3931	isc-miR-1	isc-miR-1	isc-miR-3931	isc-miR-10
isc-miR-3931	isc-miR-275	isc-miR-3931	isc-miR-3931	isc-miR-2001	isc-miR-2001
isc-miR-2001	isc-miR-263a	isc-miR-2001	iri-mir-125b	isc-miR-12	isc-miR-3931
iri-mir-125b	isc-miR-87	iri-mir-125b	isc-miR-2001	isc-miR-1	iri-mir-125b
isc-miR-263a	isc-miR-2001	isc-miR-263a	isc-miR-263a	iri-mir-125b	isc-miR-263a
isc-miR-87	isc-miR-7	isc-miR-7	isc-miR-87	isc-miR-7	isc-miR-12
isc-miR-79	isc-miR-79	isc-miR-12	isc-miR-12	isc-miR-87	isc-miR-87
isc-miR-12	iri-mir-125b	isc-miR-87	isc-miR-79	isc-miR-263a	isc-miR-7
isc-miR-7	isc-miR-305	isc-miR-305	isc-miR-7	isc-miR-305	isc-miR-79
isc-miR-276	isc-miR-12	isc-miR-79	isc-miR-305	isc-miR-276	isc-miR-305
isc-miR-305	isc-miR-276	isc-miR-276	isc-miR-276	isc-miR-79	isc-miR-276
iri-mir-X2	isc-miR-184	iri-mir-X2	isc-miR-184	isc-miR-317	isc-miR-184
isc-miR-184	isc-miR-8	isc-miR-317	iri-mir-X2	iri-mir-X2	isc-miR-317
isc-miR-317	isc-miR-2a	isc-miR-184	isc-miR-317	isc-miR-8	iri-mir-X2
iri-mir-X18	iri-mir-X2	isc-miR-8	isc-miR-8	isc-miR-184	isc-miR-8
isc-miR-8	isc-miR-317	nDS642227_71264	iri-mir-X18	isc-miR-2a	iri-mir-X18
isc-miR-307	isc-miR-307	iri-mir-X18	isc-miR-307	iri-mir-92	isc-miR-307
isc-miR-2a	iri-mir-92	isc-miR-2a	isc-miR-2a	iri-mir-X18	isc-miR-2a
iri-mir-92	iri-mir-X18	isc-miR-307	iri-mir-92	isc-miR-307	iri-mir-92
nDS633978_31969	nDS633978_31969	iri-mir-92	nDS633978_31969	isc-miR-96	nDS863301_32418
nDS863301_32418	nDS863301_32418	nDS633978_31969	iri-mir-X1b	nDS642227_71264	nDS633978_31969
isc-miR-2b	iri-mir-X1c-1	isc-miR-2b	isc-miR-96	nDS633978_31969	isc-miR-2b
isc-miR-96	iri-mir-X1c-2	nDS863301_32418	nDS863301_32418	isc-miR-2b	nDS889344_20921
iri-mir-X1b	isc-miR-2b	nDS625977_65388	isc-miR-2b	isc-miR-5312	isc-miR-96
nDS889344_20921	isc-miR-96	isc-miR-96	iri-mir-X1e	nDS973116_21151	iri-mir-X1e
nDS720277_30341	isc-miR-315	nDS748105_49761	nDS973116_21151	nDS863301_32418	iri-mir-X1b
nDS973116_21151	nDS889344_20921	nDS973116_21151	iri-mir-X1b	nDS629760_71857	nDS973116_21151
iri-mir-X11a	nDS763323_100987	nDS720277_30341	isc-miR-5312	iri-mir-X1c-2	isc-miR-5312
isc-miR-315	nDS777850_38819	isc-miR-5312	nDS663353_42925	iri-mir-X1c-1	isc-miR-315
isc-miR-5312	nDS973116_21151	nDS857739_57334	nDS720277_30341	nDS720277_30341	nDS720277_30341
nDS663353_42925	isc-miR-5312	iri-mir-X1c-2	nDS921134_3345	nDS775456_13019	iri-mir-X1c-1
nDS777850_38819	nDS921134_3345	iri-mir-X1c-1	isc-miR-315	iri-mir-X1h	iri-mir-X1c-2
nDS656447_12101	nDS720277_30341	nDS921134_3345	iri-mir-X1c-1	nDS889344_20921	nDS921134_3345
nDS921134_3345	nDS629760_71857	nDS777850_38819	iri-mir-X1c-2	nDS663353_42925	nDS962636_5276
nDS954863_73895	iri-mir-X1h	nDS663353_42925	iri-mir-X3	iri-mir-190	nDS763323_100987
nDS785766_12009	nDS934501_94490	isc-miR-315	iri-mir-190	isc-miR-315	nDS770087_49744
iri-mir-X11b	nDS663353_42925	nDS889344_20921	nDS934501_94490	nDS625977_65388	nDS663353_42925
iri-mir-X3	nDS638407_75256	nDS775456_13019	nDS882723_56475	nDS921134_3345	iri-mir-190
nDS721252_53006	nDS671483_41819	iri-mir-X1h	nDS753381_58690	nDS908024_20238	iri-mir-X3
iri-mir-190	iri-mir-X1b	iri-mir-X3	nDS896402_54125	nDS668546_37691	nDS898661_53467
iri-mir-X1c-2	iri-mir-190	nDS898661_53467	isc-miR-133	nDS826964_27357	nDS934501_94490

miRDeep2-predicted novel miRNAs with no homology to existing miRbase miRNAs are denoted with “nDS” at the beginning of the identifier.

In reviewing the lists of the 50 most abundant miRNAs expressed in each of the 6 experimental groups, 6 miRNAs (iri-mir-X11a, nDS656447_12101, nDS954863_73895, nDS785766_12009, iri-mir-X11b, nDS721252_53006) were unique to the POWV-infected 1 hour fed salivary gland libraries (Fig. 4). 4 miRNAs were unique to the 50 most abundant miRNAs expressed in the uninfected 1 hour fed salivary gland libraries (nDS882723_56475, nDS753381_58690, nDS896402_54125, isc-miR-133), 2 miRNAs were unique to the top 50 miRNAs expressed in the POWV-infected 3 hour fed salivary glands (nDS638407_75256 and nDS671483_41819), 3 miRNAs were unique to the top 50 miRNAs expressed in the uninfected 3 hour fed salivary glands (nDS908024_20238, nDS668546_37691, nDS826964_27357), 2 miRNAs were unique to the top 50 miRNAs expressed in the POWV-infected 6 hour fed salivary glands (nDS748105_49761, nDS857739_57334), and 2 miRNAs were unique to the top 50 miRNAs expressed in the uninfected 6 hour fed salivary glands (nDS962636_5276, nDS770087_49744). A single miRNA (nDS777850_38819) from the lists of the 50 most abundant miRNAs was common to the POWV-infected 1 hour, 3 hour, and 6 hour salivary gland libraries; however, no miRNA was shared between the uninfected 1 hour, 3 hour, and 6 hour salivary gland libraries.

### Differential expression of *I. scapularis* salivary gland miRNAs in response to POWV infection

To examine the global differential expression of *I*. *scapularis* salivary gland miRNAs in response to POWV infection, read counts were normalized as reads per million. A Log_2_ fold-change and corresponding adjusted P-value (FDR) were generated for the differential expression of miRNAs from POWV-infected salivary glands versus uninfected salivary glands. Log_2_ fold-changes were derived from 4 biological replicates at each of the 3 POWV-infected tick feeding time points and from 3 biological replicates at each of the 3 uninfected tick feeding time points. miRNAs with a Log_2_ fold-change expression > |1| *and* FDR ≤ 0.1 were considered significantly differentially expressed.

**Figure 5:**
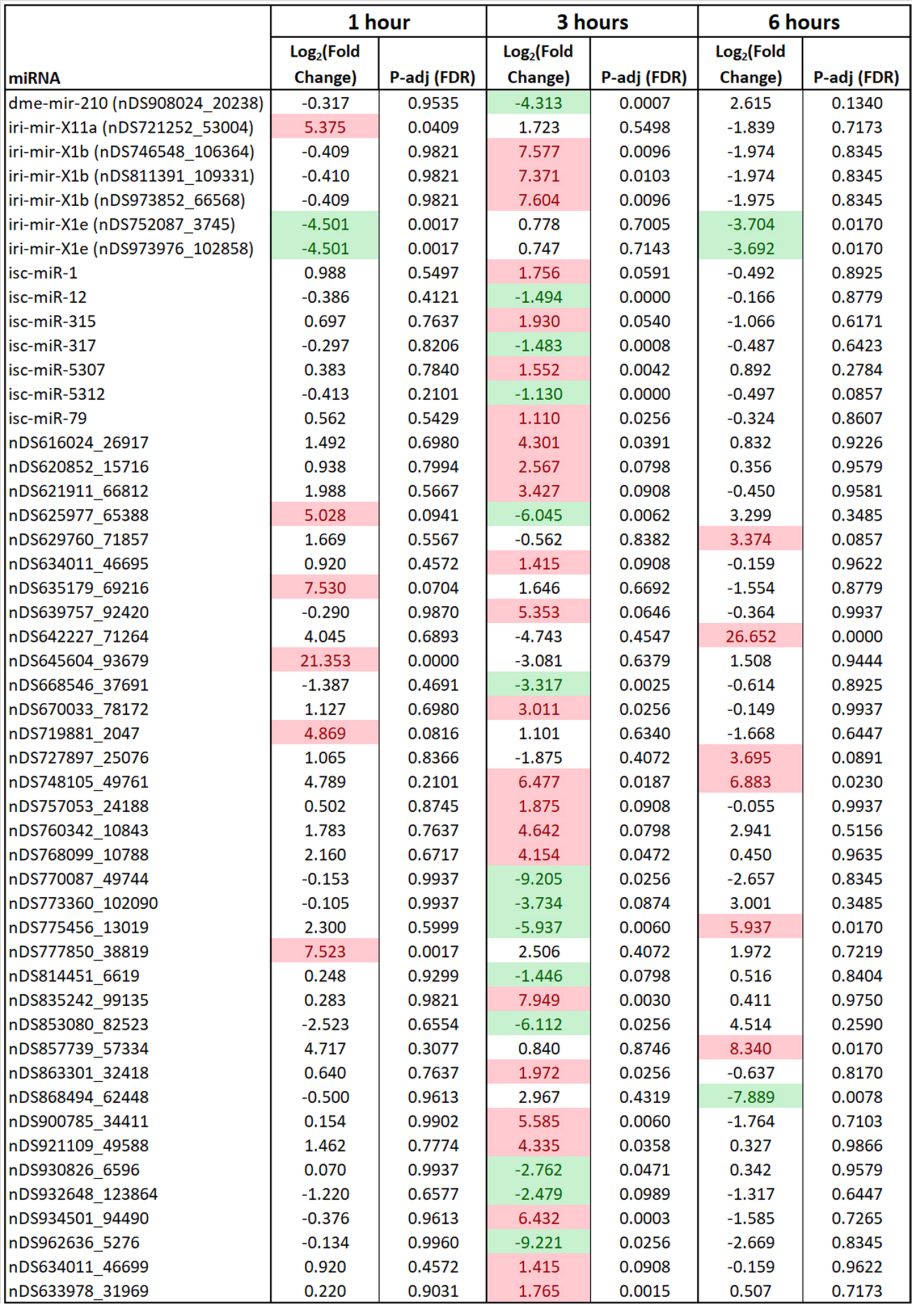
*Ixodes scapularis* salivary gland miRNAs with significant differential expression in response to POWV infection at a minimum of 1 time point. miRNAs with a Log_2_ fold-change expression > |1| *and* FDR ≤ 0.1 were considered significantly differentially expressed. Values highlighted in red indicate significant up-regulation and values highlighted in green indicate significant down-regulation.

Small RNA libraries from POWV-infected *I*. *scapularis* salivary glands showed significant alteration of miRNA expression profiles compared to the uninfected *I*. *scapularis* salivary glands after 1, 3, and 6 hours of tick feeding. 6 salivary gland miRNAs were significantly up-regulated in response to 1 hour of POWV-infected *I*. *scapularis* feeding, all of which showed Log_2_ fold change > 4 (Fig. 5). The maximum significant up-regulation was found in nDS645604_93679, with Log_2_ fold change of 21.35. nDS777850_38819, which was significantly up-regulated after 1 hour of POWV-infected tick feeding (Log_2_ fold change = 7.52), was also among the 50 most abundant miRNAs from the POWV-infected 1 hour salivary gland libraries. Two miRNAs were significantly down-regulated in response to 1 hour of POWV-infected tick feeding, both of which showed Log_2_ fold change = −4.50 (Fig. 5). nDS752087_3745, which was among the 50 most abundant miRNAs from the uninfected 1 hour salivary gland libraries, was significantly down-regulated in response to 1 hour of POWV-infected tick feeding (Log_2_ fold change = −4.50).

Across all three experimental time points, the 3 hour time point had the highest number of significantly modulated salivary gland miRNAs, 24 of which were significantly up-regulated in response to 3 hours of POWV-infected *I*. *scapularis* feeding (Fig. 5). The miRNA with maximum significant up-regulation in response to 3 hours of POWV-infected tick feeding was nDS835242_99135 (Log_2_ fold change = 7.95). 15 of the 24 significantly up-regulated 3 hour miRNAs showed Log_2_ fold change > 2. Of the 24 salivary gland miRNAs significantly up-regulated in response to 3 hours of infected tick feeding, 4 (isc-miR-1, isc-miR-315, isc-miR-5307, and isc-miR-79) matched a known previously annotated miRBase *I*. *scapularis* mature miRNA. nDS863301_32418, nDS633978_31969, nDS934501_94490, isc-miR-1, isc-miR-315, isc-miR-5307, and isc-miR-79 were significantly up-regulated at the 3 hour time point and were also among the 50 most abundant miRNAs from the POWV-infected 3 hour salivary gland libraries (Table 4 and Fig. 5). 14 salivary gland miRNAs were significantly down-regulated in response to 3 hours of POWV-infected *I*. *scapularis* feeding, and the maximum significant down-regulation was for nDS962636_5276 with a Log_2_ fold change of −9.22 (Fig. 5). 10 of the 14 significantly down-regulated 3 hour miRNAs showed Log_2_ fold change > |2|. 7 significantly down-regulated miRNAs (nDS908024_20238, isc-miR-12, isc-miR-317, isc-miR-5312, nDS625977_65388, nDS668546_37691, and nDS775456_13019) were among the 50 most abundant miRNAs from the uninfected 3 hour salivary gland libraries. Three of the significantly down-regulated miRNAs matched a known, previously annotated miRBase *I*. *scapularis* mature miRNA (isc-miR-12, isc-miR-317, and isc-miR-5312).

In response to 6 hours of POWV-infected *I*. *scapularis* feeding, 6 salivary gland miRNAs were significantly upregulated, and all 6 showed Log_2_ fold change > 3 (Fig. 5). The miRNA with maximum significant up-regulation in response to 6 hours of POWV-infected tick feeding was nDS642227_71264 (Log_2_ fold change = 26.65). 4 significantly up-regulated miRNAs (nDS642227_71264, nDS748105_49761, nDS775456_13019, and nDS857739_57334) were among the 50 most abundant miRNAs from the POWV-infected 6 hour salivary gland libraries. 3 salivary gland miRNAs were significantly down-regulated in response to 6 hours of POWV-infected *I*. *scapularis* feeding, and the maximum significant down-regulation was for nDS868494_62448 with a Log_2_ fold change of −7.89 (Fig. 5). All 3 of the significantly down-regulated 6 hour miRNAs showed Log_2_ fold change > |3|.

**Figure 6:**
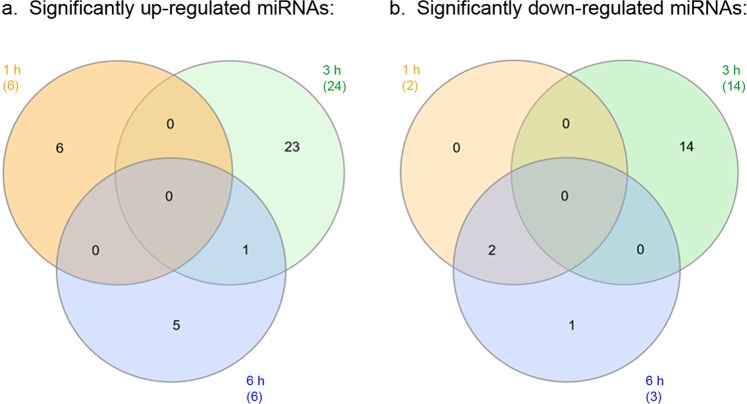
Overlap between the significantly up- and down-regulated *Ixodes scapularis* salivary gland miRNAs in response to POWV infection. The Venn diagrams show the overlap between the significantly up-regulated (**a**) or significantly down-regulated (**b**) salivary gland miRNAs after 1, 3, and 6 hours of POWV-infected tick feeding. miRNAs with a Log_2_ fold-change expression > |1| and FDR ≤ 0.1 are considered significantly differentially expressed. Numbers in parenthesis indicate the total number of miRNAs with significant differential expression at a given time point.

Upon comparing the lists of salivary gland miRNAs significantly up-regulated in response to POWV infection across the 3 experimental time points, it is evident that the majority of significantly up-regulated miRNAs were unique to each time point and not shared (Fig. 6a). No significantly up-regulated miRNA was shared between all three time points; however, a single miRNA, nDS748105_49761, was common between 3 hours and 6 hours of POWV-infected tick feeding. nDS748105_49761 was up-regulated at all three experimental time points, with significant up-regulation in response to both 3 and 6 hours of POWV-infected tick feeding (Log_2_ fold changes = 6.48 and 6.88, respectively). Analysis of the significantly down-regulated miRNAs across the 3 experimental time points also shows that the majority of miRNAs were unique to each time point and not shared between time points (Fig. 6b). None of the significantly down-regulated miRNAs were shared between all three time points. Although 14 miRNAs were significantly down-regulated in response to 3 hours of POWV-infected tick feeding, none were shared between the 1 hour and 6 hour time points. Two significantly down-regulated miRNAs (nDS752087_3745 and nDS973976_102858) were common between 1 hour and 6 hours of tick feeding.

### qRT-PCR validation of mature miRNA differential expression

**Figure 7:**
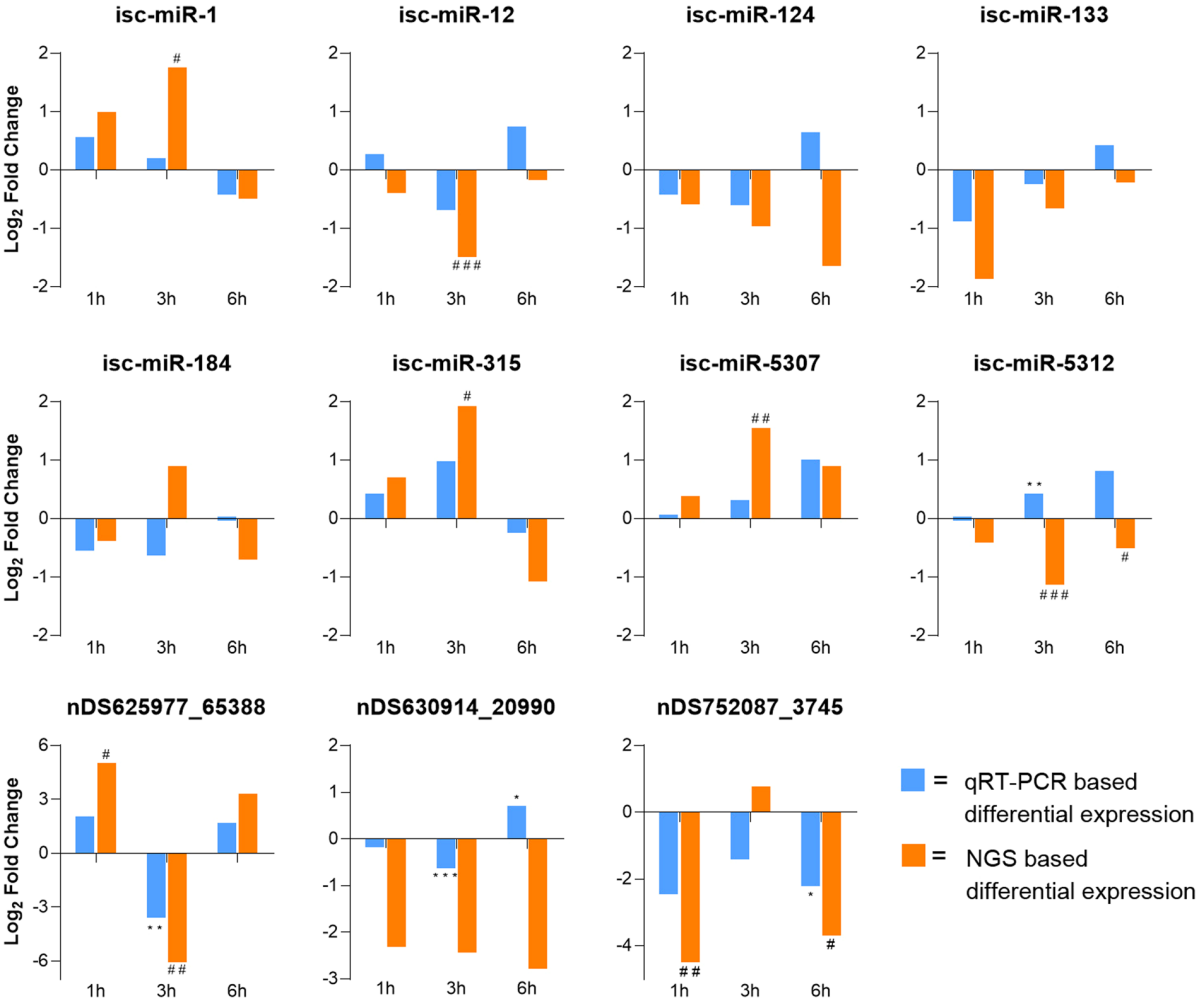
qPCR validation of select mature miRNAs that are differentially expressed in response to POWV infection. Each graph depicts the differential expression (Log_2_ fold change) of a select miRNA from POWV-infected salivary glands versus uninfected salivary glands based on NGS data (shown in orange) and qRT-PCR data (shown in blue). Significance for qRT-PCR-based differential expression is determined by the 2-tailed Student’s t test where *P < 0.05, **P < 0.01, ***P < 0.001. Significance for NGS-based differential expression is determined by a DESeq2-derived adjusted P-value (P-adj) where ^#^P-adj < 0.1, ^##^P-adj < 0.01, ^###^P-adj < 0.001.

To validate the differentially expressed miRNAs, 8 known, previously annotated *I*. *scapularis* miRNAs and 3 miRDeep2-predicted novel *I*. *scapularis* miRNAs were selected for qRT-PCR validation. For every salivary gland RNA sample that underwent small RNA library preparation, a portion of the RNA sample was converted to cDNA. Mature miRNAs were selectively converted into cDNA, and a miRNA-specific qRT-PCR reaction was performed for each sample. The qRT-PCR patterns of differential expression matched the NGS patterns in the majority of evaluated miRNAs across the three experimental time points (Fig. 7). Although it is not surprising to find discrepancies between these two methods of quantifying miRNA expression^33^, in 24 out of 33 instances (72.7%), the pattern of miRNA regulation was the same between the NGS data and the qRT-PCR data (ie. both NGS and qRT-PCR datasets were up-regulated or both datasets were down-regulated). The qRT-PCR validation data for the 8 known *I*. *scapularis* miRNAs showed that in 17 out of 24 instances the trend for the NGS data matched that of the qRT-PCR data (Fig. 7). Discrepancies were found for isc-miR-12 at 1 and 6 hours, isc-miR-124 at 6 hours, isc-miR-133 at 6 hours, isc-miR-184 at 3 hours, and isc-miR-5312 at 3 and 6 hours. Of these discrepancies, the only miRNA with significant differential expression in both the NGS and qRT-PCR datasets was isc-miR-5312 at 3 hours. The qRT-PCR Log_2_ fold change expression values for isc-miR-184 at 6 hours and isc-miR-5312 at 1 hour were very small (Log_2_ fold change = −0.0063 and −0.0031, respectively), and although these values do not clearly appear on the graphs, these patterns of differential expression matched the down-regulation pattern shown for the NGS data. The qRT-PCR validation data for the 3 miRDeep2-predicted novel *I*. *scapularis* miRNAs showed that in 7 out of 9 instances the trend for the NGS data matched that of the qRT-PCR data (Fig. 7). Discrepancies between the patterns of differential expression were found for nDS630914_20990 at 6 hours and nDS752087_3745 at 3 hours. For the 6 hour qRT-PCR versus NGS comparison of nDS630914_20990, the unmatched pattern of differential expression is likely due to the non-significant NGS data point. Overall, the qRT-PCR validation confirmed that all 11 selected miRNAs were detected in the POWV-infected and uninfected *I*. *scapularis* salivary glands and that the qRT-PCR patterns of differential expression matched the NGS patterns in the majority of evaluated miRNAs.

### *I. scapularis* salivary gland miRNAs regulate POWV replication in mammalian cells

**Figure 8:**
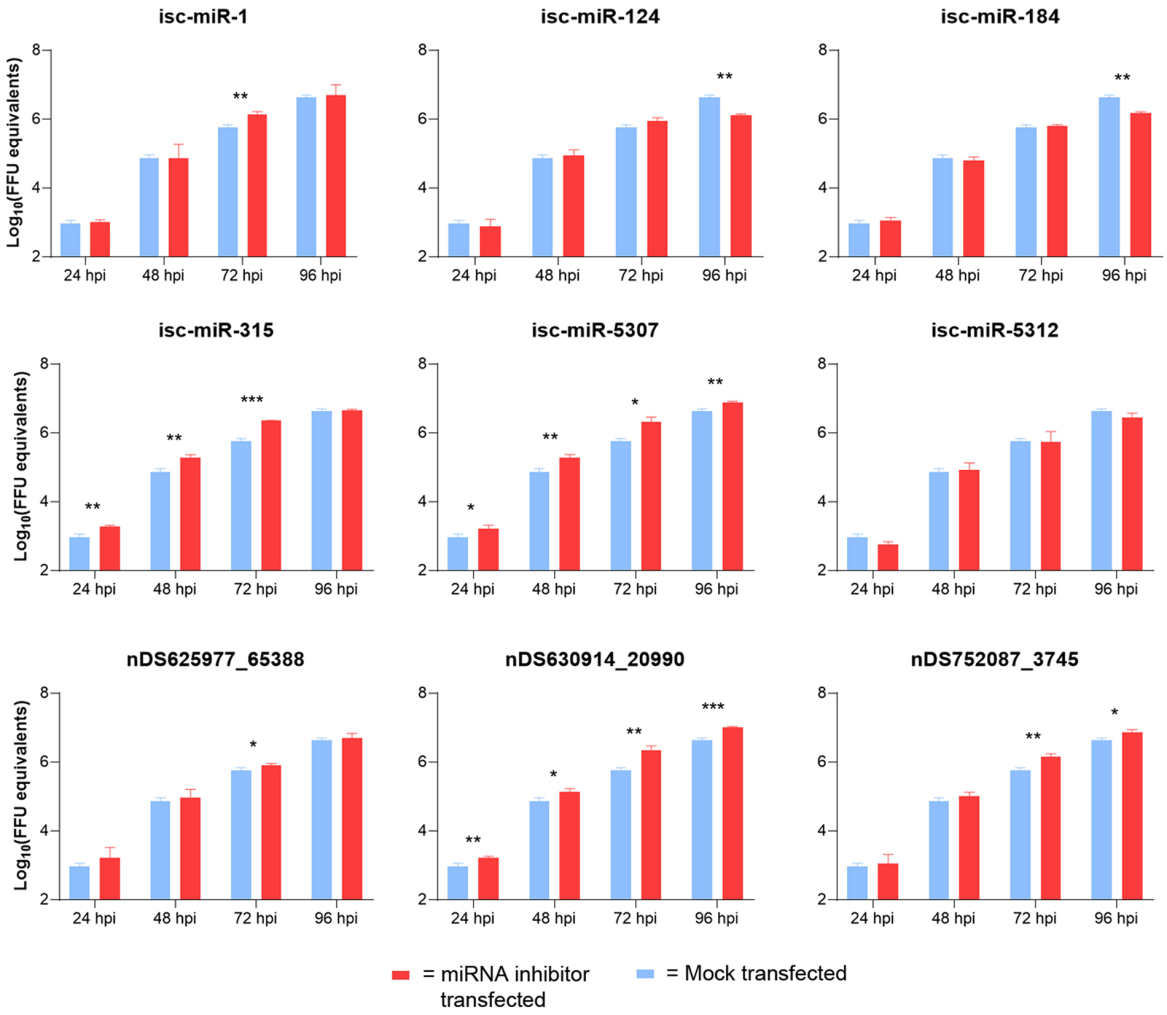
*Ixodes scapularis* saliva miRNAs regulate POWV replication in mammalian cells. VeroE6 cells were transfected with a miRNA inhibitor or mock-transfected with Lipofectamine only. 24 hours post-transfection, cells were infected with POWV (MOI = 0.05). Supernatant was collected daily for 96 hours and viral titers for each timepoint were determined via quantitative real-time PCR analysis. A 2-tailed Student’s T-test was used to analyze the significance of POWV titer differences between miRNA-transfected versus mock-transfected VeroE6 cells at each time point where *P < 0.05, **P < 0.01, ***P < 0.001.

To investigate the potential role of *I*. *scapularis* salivary gland miRNAs in POWV replication, miRNA inhibitors were transfected into VeroE6 cells to profile POWV replication in mammalian cells over time. *In vitro* data from testing 9 miRNA inhibitors demonstrated their role in regulating POWV infection. POWV titers in mock-transfected cells peaked at 96 hpi (hours post infection) at 6.63 Log_10_ FFU equivalents. At 24, 48, and 72 hpi POWV titers were significantly higher (p < 0.05) in cells transfected with isc-miR-315, isc-miR-5307, and nDS630914_20990 inhibitors (Fig. 8) than in mock-transfected cells. At 72 hpi, cells transfected with isc-miR-1, nDS625977_65388, and nDS752087_3745 inhibitors displayed significantly higher (P < 0.05) POWV titers than mock-transfected cells, and at 96 hpi, POWV titers were significantly higher in cells transfected with isc-miR-5307, nDS630914_20990, and nDS752087_3745 inhibitors compared to mock-transfected cells. In this *in vitro* study, there were two instances in which the POWV titers were significantly lower in response to miRNA inhibitor transfection compared to mock transfection. At 96 hpi, cells transfected with either isc-miR-124 or isc-miR-184 inhibitors displayed significantly (P < 0.05) lower POWV titers (6.11 Log_10_ FFU equivalents and 6.17 Log_10_ FFU equivalents, respectively) compared to mock-transfected cells (6.63 Log_10_ FFU equivalents). Though the titer differences were not significant, cells transfected with the isc-miR-5312 inhibitor displayed lower POWV titers at 24, 72, and 96 hpi than mock-transfected cells. There were no significant differences in POWV titers in non-transfected cells (POWV only) versus mock transfected cells (cells treated with RNAiMax transfection reagent in the absence of a miRNA inhibitor).

## Discussion

The complex assortment of molecules in tick saliva reflects the multifaceted host defense mechanisms and enables ticks to remain attached to the host, to successfully complete blood feeding, and to evade the host immune response^16,34,35^. Through saliva-assisted transmission (SAT), bioactive tick salivary factors modulate various host defense mechanisms, creating an environment that is more favorable for pathogen transmission and establishment. The phenomenon of SAT has been demonstrated for several TBFVs. Tick saliva is composed of hundreds of proteins that are differentially expressed over the course of the tick’s lengthy feeding process^34^. Many studies have demonstrated that various tick saliva proteins modulate host hemostasis, wound repair, and the innate and adaptive host immune responses^14^; however, few non-proteinaceous tick salivary gland molecules have been identified. Prostaglandin was the first identified non-protein component of tick saliva^36^ and was later found to have immunomodulatory properties^37^. Non-proteinaceous molecules such as prostaglandin E2, prostacyclin, purine nucleoside adenosine, endocannabinoids, and related fatty acids have all been shown to be immunomodulatory components of tick saliva^37–39^.

miRNAs are yet another category of non-proteinaceous molecules that have only recently been detected in the saliva and salivary glands of several tick species^23,24,27^. These short, ~22 nucleotide, non-coding RNAs are involved in post-transcriptionally regulating host target genes in various physiological and pathological processes of eukaryotes. Detection of *I*. *ricinus* saliva-specific miRNAs provided the first evidence of tick salivary miRNAs that regulate host gene expression^27^. Unlike tick salivary gland proteins, no immune response can be mounted against these non-proteinaceous miRNA molecules. By gaining an understanding of how tick miRNAs immunomodulate the tick-host interface in the presence of a pathogen, we can build a foundation for developing tick miRNA-based therapeutic candidates.

In this study, we present the first data on tick salivary gland miRNA expression profiles in relation to virus infection and transmission. The tick miRNA profiles in *I*. *scapularis* salivary glands were composed of two peaks, including reads of 21–23 nt and reads of 26–29 nt (Fig. 1). The presence of these two peaks is consistent with previous reports of small RNA sequencing in other tick and mosquito miRNA studies. After 1, 3, and 6 hours of tick feeding, the size distribution peaks at 21 and 22 nt were greater for the POWV-infected salivary glands than for the uninfected salivary glands, suggesting that there is more abundance of miRNAs in the POWV-infected tick salivary glands compared to the uninfected salivary glands. Although the present study focused on *I*. *scapularis* miRNAs (21–23 nt), it would be beneficial for a future study to examine the potential role of other small RNA populations in regulating viral replication and transmission during tick feeding.

Using miRDeep2 prediction software, various novel tick miRNAs were predicted from the *I*. *scapularis* salivary gland libraries. The presence of select novel predicted miRNAs (nDS625977_65388, nDS630914_20990, and nDS752087_3745) was confirmed via their detection with qRT-PCR (Fig. 7). Additional validation of other predicted novel miRNAs is needed, especially for those predicted miRNAs with high miRDeep2 probability scores (Supplemental Table 1). The top 50 most abundant miRNAs were compared for each of the 6 experimental groups. 39 miRNAs were shared between all 6 groups (Fig. 4), of which 28 matched a known, previously annotated miRBase *I*. *scapularis* mature miRNA and 5 matched a previously annotated *I*. *ricinus* miRNA. Previous reports indicate that evolutionarily-conserved miRNAs are usually highly expressed^23,24^. Since these shared, highly expressed miRNAs were detected in both the uninfected and POWV-infected tick salivary glands, the majority are likely constitutively expressed *I*. *scapularis* salivary gland miRNAs that are not involved in regulating POWV infection.

In *I*. *scapularis* female ticks, infection with POWV altered the salivary gland miRNA profile, with multiple instances of temporal variation in miRNA expression observed between 1, 3, and 6 hours of tick feeding. NGS analysis identified novel *I*. *scapularis* salivary gland miRNAs as well as known, previously annotated miRNAs that were differentially expressed upon POWV infection (Fig. 5). At each time point, more miRNAs showed significant up-regulation compared to down-regulation in response to POWV-infected tick feeding. The following 7 known *I*. *scapularis* miRNAs were significantly up- or down-regulated in response to POWV-infected tick feeding: isc-miR-1, isc-miR-12, isc-miR-315, isc-miR-317, isc-miR-5307, isc-miR-5312, and isc-miR-79.

Although no previous study has examined tick miRNAs in relation to pathogen infection and transmission, lipopolysaccharide (LPS)-induced differential expression of miRNAs in *R*. *haemaphysaloides* ticks has been documented. miR-1 and miR-315 were in the top 10 most abundant miRNAs in PBS- and LPS-injected *R*. *haemaphysaloides* ticks, but neither met the cut-off for significant up- or down-regulated expression in response to LPS^26^. miR-79-3p was significantly down-regulated in female *R*. *haemaphysaloides* ticks after LPS injection^26^. In the present study, isc-miR-1, isc-miR-315, and isc-miR-79 were all significantly up-regulated in response to 3 hours of POWV-infected tick feeding (Fig. 5). Discrepancies in the patterns of LPS-induced versus POWV-induced differential expression for miR-1, miR-315, and miR-79 could be attributed to the following: different types of samples processed (ie. individual tick salivary glands from blood-fed POWV-infected ticks versus whole tick bodies from LPS-injected unfed ticks), specific pathogen-tick species interactions, the physiology of unfed ticks versus feeding ticks.

Recently, NGS of uninfected adult *I*. *ricinus* saliva-derived libraries provided the first evidence of miRNAs present in pure tick saliva, and molecular indicators suggest that that these saliva-derived miRNAs are secreted in exosomes^27^. Several of the same known (miRBase annotated) miRNAs that were significantly modulated in *I*. *scapularis* female salivary glands in response to POWV-infected tick feeding were also detected in the salivary glands and saliva of uninfected feeding *I*. *ricinus* females (miR-1, miR-12, miR-317, and miR-5307). In the *I*. *ricinus* study, iri-miR-5307-3p was identified as a putative feeding-regulated miRNA because its expression levels increased as tick feeding progressed^27^, and in the present study, isc-miR-5307 was significantly up-regulated in response to 3 hours of POWV-infected *I*. *scapularis* feeding (Fig. 5). In uninfected *I*. *ricinus* females, iri-miR-317-3p was 249-fold overrepresented in saliva but virtually absent in the salivary glands^27^. This study showed that in response to 3 hours of POWV-infected *I*. *scapularis* feeding, isc-miR-317 and isc-miR-5312 were significantly down-regulated. Interestingly, rmi-miR-317 and rmi-miR-5312 were significantly down-regulated when *R*. *microplus* larvae exposed to a host for six hours (but not allowed to feed) were compared to unexposed larvae, providing evidence that host-odor recognition triggers changes in rmi-miR-317 and rmi-miR-5312 expression^23^.

Prior to this publication, only one other study had characterized arthropod salivary miRNA profiles in response to virus infection. Exogenous miRNAs were identified in *A*. *aegypti* and *A*. *albopictus* mosquito saliva, several of which were expressed only upon CHIKV infection^29^. The patterns of virus-induced differential expression between POWV-infected *I*. *scapularis* salivary gland miRNAs and CHIKV-infected mosquito saliva miRNAs aligned in several instances. For example, isc-mir-315 was significantly up-regulated in response to 3 hours of POWV-infected tick feeding, corresponding with the highly up-regulated expression levels of aae-miR-315 in CHIKV-infected *A*. *aegypti* and *A*. *albopictus* saliva^29^. The significant down-regulation of isc-miR-317 in 3 hour-fed POWV-infected *I*. *scapularis* salivary glands is complemented by aae-miR-317 down-regulation in CHIKV-infected *A*. *albopictus* saliva^29^. Additionally, miR-317-5p was significantly down-regulated in pools of whole *A*. *aegypti* and *A*. *albopictus* mosquitoes infected with Dengue virus 2^40,41^. Aae-miR-1-5p was significantly up-regulated in whole *A*. *aegypti* that were 7 days post-Zika virus infection^33^, an expression pattern that was mirrored, albeit at different timelines post-infection, with significant up-regulation of isc-miR-1 in 3 hour fed POWV-infected *I*. *scapularis* salivary glands.

Our *in vitro* data from testing 9 miRNA inhibitors demonstrated their potential role in regulating POWV infection (Fig. 8). POWV titers were significantly decreased in mammalian cells at 96 hpi when isc-miR-124 and isc-miR-184 were inhibited, suggesting that the presence of these miRNAs in tick salivary glands (and potentially in tick saliva) could enhance POWV infection and later dissemination from the cutaneous site of infection. In contrast, for several of the miRNAs inhibited, such as isc-miR-315, isc-miR-5307, miR-nDS630914_20990, and miR-nDS752087_3745, there were significant increases in POWV titers for at least two time points examined. Because inhibiting some miRNAs resulted in increased POWV titers in our *in vitro* assay, certain miRNAs present in tick saliva could potentially limit POWV replication and transmission to a host. Our data demonstrates that during the initial hours of tick feeding, unique expression patterns of specific tick salivary gland miRNAs are detected. Specifically, 52 salivary gland miRNAs were significantly differentially expressed in response to 1, 3, or 6 hours of POWV-infected tick feeding. It is plausible that specific tick salivary gland-derived miRNA signatures could temporally regulate POWV replication in certain host tissues, and it is the subject of our future study.

Now that virus-induced differential expression has been identified for multiple tick and mosquito salivary miRNAs, it will be beneficial to determine the functional role of these miRNAs in the transmission and establishment of infection during vector blood feeding. This process has begun for mosquito miRNAs, and a recent comprehensive review outlined the miRNAs with validated functions in regulating West Nile virus, Dengue virus, and CHIKV infection^20^. The *I*. *scapularis* salivary gland miRNAs that displayed significant differential expression upon POWV-infected tick feeding will be the subject of our future *in vivo* studies. By gaining an understanding of how non-proteinaceous tick saliva factors such as miRNAs immunomodulate the tick-host interface in the presence of a virus, we will build a foundation for developing potential tick saliva-based therapeutic candidates.

## Methods

### Ethics statement

All experiments involving mice and infected ticks were conducted in arthropod containment level 3 (ACL-3) facilities in strict accordance with an animal use protocol approved by the University of Texas Medical Branch (UTMB) Institutional Animal Care and Use Committee (IACUC: # 0907054).

### Cells and viruses

African green monkey kidney (VeroE6) cells were purchased from the American Type Culture Collection (ATCC) and maintained in culture with Modified Eagle’s Medium (MEM) supplemented with 10% fetal bovine serum, 1% non-essential amino acids, and a 1% antibiotic mixture of penicillin/streptomycin incubated at 37 °C with 5% CO_2_. The World Reference Center for Emerging Viruses and Arboviruses at UTMB provided stock of the prototypic LB strain of POWV, which had previously been passaged 7 times in suckling mice brains. The stock was then passaged 6 times on VeroE6 cells. Stock virus titers were determined by focus-forming immunoassay as described previously^42^.

### Animals

Five-week-old female BALB/cj mice were received from The Jackson Laboratory (Bar Harbor, ME). Mice were allowed to acclimate to the local environment before incorporation into the experiments, at which point the mice were six weeks of age.

### Tick infection and infestation on mice

Uninfected adult *Ixodes scapularis* ticks were maintained in our lab within the UTMB arthropod containment level 2 (ACL-2) facilities. Tick vials were stored inside an incubator at 22 °C and 90–93% relative humidity. The incubator photoperiod was set on a 16:8 hour cycle. Adult, female *I*. *scapularis* were synchronously infected with POWV-LB strain (7 suckling mouse brain passages followed by 5 Vero cell passages) as previously described^43^. Control female ticks were mock-infected with media. POWV-infected and mock-infected *I*. *scapularis* females were co-housed with uninfected male *I*. *scapularis* and stored inside a desiccator at 22 °C to allow POWV replication. At 40 days post-synchronous infection, the *I*. *scapularis* females were infested on mice. Viral infection in each pair of tick salivary glands was confirmed by q-RT-PCR on a CFX96 real-time PCR (BioRad) using 10 µM POWV-specific forward and reverse primers (Table 5) and the iTaq Universal SYBR Green One-Step kit (BioRad). The following cycling protocol was used: 10 minutes at 50 °C; 1 minute at 95 °C; 10 seconds at 95 °C followed by 30 seconds at 60 °C for 45 cycles; and an 81-cycle (+0.5 °C/cycle) 55–95 °C melt curve.

**Table 5:** Primer sequences for qPCR primers.

Primer name	Sequence (5′ - 3′)
isc-miR-1	TGGAATGTAAAGAAGTATGGAG
isc-miR-12	TGAGTATTACATCAGGTACTGGT
isc-miR-124	TAAGGCACGCGGTGAATGCCAAG
isc-miR-133	TTGGTCCCCTTCAACCAGCTGT
isc-miR-184	TGGACGGAGAACTGATAAGGGC
isc-miR-315	TTTTGATTGTTGCTCAGAAGGC
isc-miR-5307	TAATCTCATTTGGTATCTCTGGG
isc-miR-5312	TGGCTGAACGTTGTTATGCGT
miR-DS630914_20990	GGTGTTTGGTTGGAACTCGTG
miR-DS752087_3745	GCTGTTAGTTTGTGGGTTGGT
miR-DS625977_65388	ATGTGTGCCGGCCTTACATGAA
RNU6-2	CGCAAGGATGACACGCAAATTCGTGAAGCGTTCCATATTTTT
POWV-F	CCGAGCCAAAGTGAGGATGT
POWV-R	TCTTTTGCCGAGCTCCACTT

Tick containment capsules were prepared as previously described^43^. Two days prior to tick infestation, mice were anesthetized with isoflurane and the torsos were shaved. One capsule was adhered to the dorsum of each mouse using livestock Kamar adhesive (Kamar Inc., Steamboat Springs, CO). On the day of tick infestation, a single *I*. *scapularis* female was placed inside each capsule and allowed to feed on the mouse for 1, 3, or 6 hours. At the experimental time points, mice were euthanized with CO_2_ inhalation according to IACUC protocols. The feeding tick was removed and tick salivary glands were dissected in sterile phosphate-buffered saline (PBS) on a sterile microscope slide. Each pair of salivary glands was stored in a 100 µL aliquot of QIAzol lysis reagent (Qiagen).

### RNA extractions from tick salivary glands

Tick salivary glands were homogenized in QIAzol reagent using a motorized hand-held pestle. An additional 600 µL of QIAzol reagent was added to each homogenized salivary gland sample. RNA extractions were performed as outlined in the miRNeasy Mini kit handbook (Qiagen), resulting in purification of total RNA, including small RNAs. Viral infection in tick salivary glands was confirmed by quantitative real-time PCR (qPCR) using primers specific to the NS5 gene of POWV as previously described^17^.

### Next Generation Sequencing

Small RNA libraries were made using the NEBNext smallRNA Multiplex kit (New England Biolabs, In.) following the manufacturer’s protocol. Briefly, short adapter oligonucleotides are ligated to each end of the small RNAs in the sample, a cDNA copy is made with reverse transcriptase, and PCR is used to add sample specific barcodes and Illumina sequencing adapters.

The final concentration of all NGS libraries was determined using a Qubit fluorometric assay and the DNA fragment size of each library was assessed using a DNA 1000 high-sensitivity chip on an Agilent 2100 Bioanalyzer. After purification by polyacrylamide gel-electrophoresis, the sample libraries were pooled and sequenced on an Illumina NextSeq550 (single end 75 base) using TruSeq SBS kit v3 (Illumina) and protocols defined by the manufacturer.

### Data analysis

The miRDeep2 software package, version 2.0.0.8, was used to process the sequencing data. For the novel miRNA prediction step, the reads from all the samples were combined. The mapper function of miRDeep2 first trims the adapter sequences from the reads and converts the read files from fastq to fasta format. Reads shorter than 18 bases were discarded. These reads were then mapped to the *Ixodes scapularis* reference sequence using the default miRDeep2 mapper function parameters. For the final miRDeep2 steps, the trimmed reads were mapped to the known *Ixodes scapularis* miRNAs from the miRBase database (Version 22) and quantified. Next, the program used the reads previously mapped to the *I*. *scapularis* reference to predict potential novel miRNAs. The output file included a score reflecting the likelihood that the predicted miRNA is real, the sequence and location of the possible miRNA, and the number of reads that mapped to it. A total of 323 unique novel miRNAs had a miRDeep2 score of 4 or greater.

For differential expression analysis, reads from each sample were individually mapped to the *I*. *scapularis* reference with bowtie (version 1.2.2, parameters: -v2 –l 18 –a –M 10 –best –strata) and reads mapping to the known and novel miRNAs were quantified with featureCounts version 1.6.2^44,45^. Differential expression of miRNAs in salivary glands upon POWV infection was analyzed using DESeq2, version 1.20.0, following the DESeq2 vignette^46^.

### Validation of mature miRNAs by qRT-PCR

The miScript II RT kit (Qiagen) was used to convert RNA samples to cDNA. Mature miRNAs were selectively converted into cDNA by using the kit’s miScript HiSpec Buffer for the reverse transcription reaction. 4 µL of RNA was used per 20 µL reverse transcription reaction. An additional reaction was prepared with no RNA template. The miScript II RT reaction was heated on a Mastercycler-Pro S thermos cycler (Eppendorf). The cDNA was diluted by adding 40 µL of nuclease-free water to bring the final cDNA volume to 60 µL. Real-time PCR was performed with a miScript SYBR Green PCR kit (Qiagen), using the kit’s proprietary-sequence universal primer as the reverse primer and 10 µM of a miRNA-specific forward primer (Table 5). 2 µL of cDNA was used per PCR reaction, and the volumes of master mix and primer used were those recommended by the manufacturer. 25 µL reactions were loaded into the wells of a 96-well plate to create customized miScript PCR arrays. Each real-time PCR array was run on a CFX96 real-time PCR (BioRad) with the following cycle: 95 °C for 15 minutes, followed by forty cycles of 94 °C for 15 seconds, 55 °C for 30 seconds, and 70 °C for 30 seconds. The ramp rate was set at 1 °C/second. The iCycler’s software was used to calculate the Ct values for all analyzed miRNAs. The ΔΔCt method was used to calculate fold-changes in gene expression between test groups (POWV-infected tick salivary glands) and control groups (uninfected tick salivary glands). The miRNA expression for each sample was normalized to the expression of RNU6-2 small nuclear RNA (Qiagen). Statistical significance was calculated by the 2-tailed Student’s T-test comparing the miRNA expression in POWV-infected salivary glands versus uninfected salivary glands. Significant values were those where P < 0.05.

### *In vitro* miRNA inhibition assay

The following miRNAs were selected for inclusion in the *in vitro* miRNA inhibition assay: isc-miR-1, isc-miR-124, isc-miR-184, isc-miR-315, isc-miR-5307, isc-miR-5312, isc-miR-nDS625977_65388, isc-miR-nDS630914_20990, and isc-miR-nDS752087_3745. The miRNA inhibitors were designed based on the miRNA sequences (Table 6) and synthesized by Integrated DNA Technologies. VeroE6 cells were used for this *in vitro* miRNA inhibition assay as they are an epithelial cell line, and fibroblasts are an early skin cell target of POWV at the tick feeding site^12^. 24-well plates of VeroE6 cells were seeded at 5 × 10^4^ cells/well. 24 hours post-seeding, the VeroE6 cells were transfected in triplicate with 55 pmol of each miRNA inhibitor via Lipofectamine RNAiMax transfection reagent (ThermoFisher Scientific). As a control, cells were mock-transfected without a miRNA inhibitor. 24 hours post-transfection, cells were infected with POWV at a multiplicity of infection of 0.05. Mock-transfected cells were also infected with POWV. Cell supernatant aliquots of 50 µL were harvested from each replicate every 24 hours until 96 hours post-infection, and 50 µL of fresh infection media was added to each well to replace the sample volume.

**Table 6:** miRNA inhibitor sequences.

miRNA inhibitor name	Sequence
isc-miR-1-inhibitor	mC/ZEN/mUmCmCmAmUmAmCmUmUmCmUmUmUmAmCmAmUmUmCmC/3ZEN/
isc-miR-124-inhibitor	mC/ZEN/mUmUmGmGmCmAmUmUmCmAmCmCmGmCmGmUmGmCmCmUmU/3ZEN/
isc-miR-184-inhibitor	mG/ZEN/mCmCmCmUmUmAmUmCmAmGmUmUmCmUmCmCmGmUmCmC/3ZEN/
isc-miR-315-inhibitor	mG/ZEN/mCmCmUmUmCmUmGmAmGmCmAmAmCmAmAmUmCmAmAmA/3ZEN/
isc-miR-5307-inhibitor	mC/ZEN/mCmCmAmGmAmGmAmUmAmCmCmAmAmAmUmGmAmGmAmUmU/3ZEN/
isc-miR-5312-inhibitor	mA/ZEN/mCmGmCmAmUmAmAmCmAmAmCmGmUmUmCmAmGmCmC/3ZEN/
isc-miR-DS625977_65388-inhibitor	mU/ZEN/mUmCmAmUmGmUmAmAmGmGmCmCmGmGmCmAmCmAmCmA/3ZEN/
isc-miR-DS630914_20990-ihibitor	mC/ZEN/mAmCmGmAmGmUmUmCmCmAmAmCmCmAmAmAmCmAmC/3ZEN/
isc-miR-DS752087_3745-inhibitor	mA/ZEN/mCmCmAmAmCmCmCmAmCmAmAmAmCmUmAmAmCmAmG/3ZEN/

Each cell supernatant sample was mixed with TRIzol LS reagent (ThermoFisher Scientific), and viral RNA extractions were performed using a combination of TRIzol LS and QIAamp Viral RNA mini (Qiagen) protocols as previously described^17^. Virus titer for each cell supernatant sample was determined by q-RT-PCR with the parameters outlined above for the tick salivary gland viral load analysis. Briefly, POWV genomic RNA was assayed using POWV-specific forward and reverse primers (Table 5) for q-RT-PCR. RNA was extracted from a POWV sample of known titer (expressed in focus-forming units), and serial dilutions were used to generate a standard curve for viral load quantification as previously described^17^. Ct values for each supernatant sample were converted to focus-forming unit equivalents based on the in-house generated standard curve. A 2-tailed Student’s T-test was used to analyze the significance of POWV titer differences between miRNA inhibitor-transfected versus mock-transfected VeroE6 cells at each time point. Significant values were those where P < 0.05.

## Supplementary information

Supplemental Table 1
